# Author Correction: Coherent correlation imaging for resolving fluctuating states of matter

**DOI:** 10.1038/s41586-023-06224-z

**Published:** 2023-05-24

**Authors:** Christopher Klose, Felix Büttner, Wen Hu, Claudio Mazzoli, Kai Litzius, Riccardo Battistelli, Sergey Zayko, Ivan Lemesh, Jason M. Bartell, Mantao Huang, Christian M. Günther, Michael Schneider, Andi Barbour, Stuart B. Wilkins, Geoffrey S. D. Beach, Stefan Eisebitt, Bastian Pfau

**Affiliations:** 1 Max Born Institute, Berlin, Germany; 2 Department of Materials Science and Engineering, Massachusetts Institute of Technology, Cambridge, MA USA; 3 National Synchrotron Light Source II, Brookhaven National Laboratory, Upton, NY USA; 4 Helmholtz-Zentrum für Materialien und Energie, Berlin, Germany; 5 IV Physical Institute, University of Göttingen, Göttingen, Germany; 6 Zentraleinrichtung Elektronenmikroskopie (ZELMI), Technische Universität Berlin, Berlin, Germany; 7 Institut für Optik und Atomare Physik, Technische Universität Berlin, Berlin, Germany

**Keywords:** Magnetic properties and materials, Imaging techniques, Magnetic properties and materials

Correction to: *Nature* 10.1038/s41586-022-05537-9 Published online 18 January 2023

In the version of this article originally published, Sergey Zayko was missing from the author list and has now been included in the HTML and PDF versions of the article.

