# Supplementary figures and images for: Coherent correlation imaging for resolving fluctuating states of matter
Source: Nature. 2023 Jan 18;614(7947):256–61. doi: 10.1038/s41586-022-05537-9 (PMC9908557; doi:10.1038/s41586-022-05537-9)

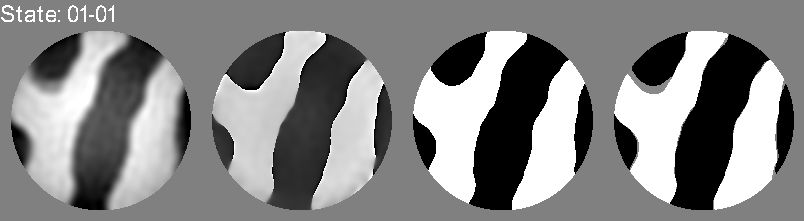

Supplement: Supplementary file 3 — Supplementary Video 2 [file 41586_2022_5537_MOESM3_ESM.gif]
